# Immunochemical characterization of polysaccharide antigens from six clinical strains of Enterococci

**DOI:** 10.1186/1471-2180-6-62

**Published:** 2006-07-12

**Authors:** Carolyn T Hsu, Amanda L Ganong, Barbara Reinap, Zafiria Mourelatos, Johannes Huebner, Julia Y Wang

**Affiliations:** 1 Channing Laboratory, Department of Medicine, Brigham and Women's Hospital, Harvard Medical School, Boston, MA02115, USA; 2 Division of Infectious Diseases, Department of Medicine, University Hospital Freiburg, Germany

## Abstract

**Background:**

Enterococci have become major nosocomial pathogens due to their intrinsic and acquired resistance to a broad spectrum of antibiotics. Their increasing drug resistance prompts us to search for prominent antigens to develop vaccines against enterococci. Given the success of polysaccharide-based vaccines against various bacterial pathogens, we isolated and characterized the immunochemical properties of polysaccharide antigens from five strains of *Enterococcus faecalis *and one strain of vancomycin-resistant *E. faecium*.

**Results:**

We cultured large batches of each strain, isolated sufficient quantities of polysaccharides, analyzed their chemical structures, and compared their antigenic specificity. Three classes of polysaccharides were isolated from each strain, including a polyglucan, a teichoic acid, and a heteroglycan composed of rhamnose, glucose, galactose, mannosamine, and glucosamine. The polyglucans from all six strains are identical and appear to be dextran. Yields of the teichoic acids were generally low. The most abundant polysaccharides are the heteroglycans. The six heteroglycans are structurally different as evidenced by NMR spectroscopy. They also differ in their antigenic specificities as revealed by competitive ELISA. The heteroglycans are not immunogenic by themselves but conjugation to protein carriers significantly enhanced their ability to induce antibodies.

**Conclusion:**

The six clinical strains of enterococci express abundant, strain-specific cell-surface heteroglycans. These polysaccharides may provide a molecular basis for serological typing of enterococcal strains and antigens for the development of vaccines against multi-drug resistant enterococci.

## Background

Enterococci are part of the normal flora of the gastrointestinal and genitourinary tracts and colonize the bowels of more than 90% of healthy humans [1]. Enterococci are opportunistic pathogens with generally low virulence, but their intrinsic and increasing acquired resistance to multiple drugs have made them a major cause of nosocomial infections [2,3]. Enterococci are intrinsically resistant to a number of antimicrobial drugs, including cell wall-active agents, many commercially available aminoglycosides, and vancomycin – the antibiotic of last resort against multidrug-resistant bacteria [4-7]. Enterococci can cause severe invasive infections such as peritonitis, endocarditis, meningitis, and abscesses in the urinary tract, along surgical sites, the lung and pleural spaces, facial sinuses, and peritoneal cavities [8,9]. Because enterococci can spread rapidly among hospital patients and transfer their antibiotic resistance genes widely, even to other bacterial species, they may cause epidemics difficult to control and thus pose a significant threat to public health. The ever-increasing resistance of enterococci to multiple drugs prompts us to search for prominent antigens for the development of vaccines.

Enterococci are Gram-positive cocci that share morphology and Lancefield antigenicity with group D streptococci. Invasive Gram-positive pathogens, such as *Streptococcus pneumoniae*, are often encapsulated with weakly immunogenic polysaccharides (PSs). These PSs are major virulence factors. It has been found that the structural differences between capsular PSs form the molecular basis of serologic specificity for many species such as *S. pneumoniae *[10]. Specific antibodies to *S. pneumoniae *PSs promote opsonization and phagocytosis and thereby confer protection against pneumococcal infection. Many successful antibacterial vaccines, such as ones against various serotypes of *S. pneumoniae*, type b of *Haemophilus influenzae*, and group C of Neisseria meningitides, have been developed based on their capsular PSs; and many other polysaccharide-based vaccines are under development [11]. PSs and PS-synthesis genes have been noted to be widespread among enterococcal strains [12-15]. Enterococcal PSs have been demonstrated to play important roles in enterococcal pathogenesis, such as conferring resistance to phagocytosis [16]. Furthermore, enterococcal PSs have been proposed as promising vaccine antigens [17-19].

Among enterococcal species, *Enterococcus faecalis *and *E. faecium *are the two major human pathogens, accounting for 85–89% and 10–15% of all enterococcal infections, respectively [8,9]. Previous investigations have revealed that enterococci express surface PSs analogous to capsules of other pathogenic bacteria [20-23]. However, only a few types of enterococcal PSs have been characterized with respect to their structure and antigenicity, and then only partially so. It is not known whether strains of enterococci express different PSs, nor how many types of PSs exist within the enterococci family. On the hypothesis that cell-surface PSs of enterococci form an antigenic basis for serotyping and are promising vaccine candidates, we investigated and compared the immunochemical properties of PSs from six clinical strains of enterococci, including five strains of *E. faecalis *and one strain of vancomycin-resistant *E. faecium*.

## Results

### Three different PSs were isolated from each enterococcal strain

To ease the PS isolation and purification, bacteria of each enterococcal strain were grown in dialyzed Columbia broth containing only nutrients with size less than 10 kDa, which were removed by dialysis later in the purification process. PSs, released by bacterial cells during fermentation, were found negligible when we attempted to isolate the PSs from the culture medium. Therefore, all PSs characterized in this study are cell-attached.

**Figure 1:**
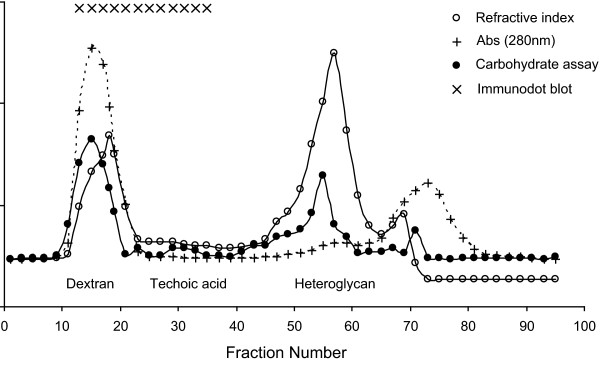
**A typical Sephacryl S400 column elution profile of the three PSs isolated from *E. faecium *B210860**. Elution profiles from the five *E. faecalis *strains are similar. Fractions positive in immunodot blotting with antiserum raised with *E. faecium *B210860 cells are indicated. The dextran fractions are contaminated with proteins as revealed by UV absorbance at 280 nm. The heteroglycan fractions are protein-free, positive in carbohydrate assay, and do not react with antisera induced by bacterial cells.

Of various enterococcal strains examined in this and our unpublished studies, three pools of PSs were obtained from each of the six enterococcal strains, eluting from the size-exclusion Sephacryl S400 column in the same order (Fig. 1). The first PS eluted close to the void volume. As determined by GC-MS analysis of its alditol acetate derivative (data not shown), this PS consists solely of glucose. Based on its 1D ^1^H and 2D ^1^H-^13^C HMQC spectra (data not shown), this PS is a poly-α-D-(1→6)-glucose, i.e., dextran. Dextran was found in all six enterococcal stains in this study and also in other enterococcal strains (unpublished results). The second PS consisted of disaccharide repeating units as suggested by NMR analysis. Because of the low yield of this PS, it was difficult to conduct further analysis. However, we speculate this PS might be consistent with the previously identified teichoic acid [24]. The third PS is a complex heteroglycan, as revealed by NMR, and its structure varies among different strains (see below). The heteroglycans were the most abundant PSs isolated from each enterococcal strain. They are relatively small, with average molecular sizes of ~20 kDa, as compared with the dextran standards.

When blotted with sera raised against whole bacterial cells, column-eluting fractions corresponding to the dextran-like PS and the teichoic acids were reactive. Because the dextan-like PS fractions were contaminated with proteins, the immune reactivity is most likely due to the contaminants but not the PS. The reactivity of teichoic acids with immune sera is consistent with previous findings [23]. The heteroglycans did not react with immune sera raised against the whole bacterial cells, indicating that these PSs are not natively immunogenic on the enterococcal cell surface.

### Chemical structural analysis of heteroglycans

Monosaccharide composition of the heteroglycans was identified by GC-MS analysis of their corresponding alditol acetate derivatives. The analysis revealed that the heteroglycans isolated from the six strains of *E. faecalis *and *E. faecium *contained the same five monosaccharides, namely, rhamnose, glucose, galactose, mannosamine, and glucosamine. Furthermore, the mannosamine and glucosamine residues were N-acetylated as suggested by NMR analysis. Although these PSs contain the same components, five monosaccharides can form an enormous number of differently structured PSs by varying their sequences and linkage sites. Therefore, we relied on NMR spectroscopy to distinguish these PSs.

**Figure 2:**
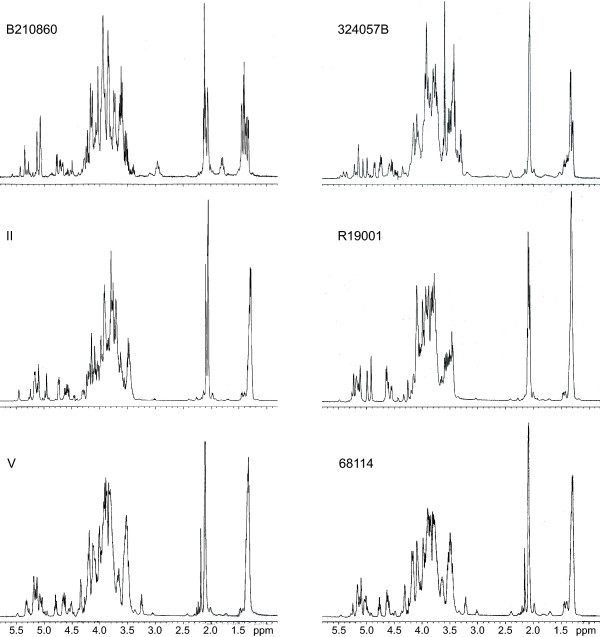
**Proton NMR spectra of the heteroglycan PSs from six enterococcal strains**. The different signal patterns indicate different structures. The PSs from strain V and 68114 have very similar spectra. Note that the signals at ~2.1 ppm are due to N- or O-acetyl groups and that the difference in intensity and pattern reveal different acetylation of each PS. Also note that the water signal at 4.45 pm has been suppressed in all spectra.

The heteroglycans from the six enterococcal strains display distinct patterns on their ^1^H-NMR spectra (Fig. 2). In each spectrum, signals between 5.6 and 4.5 ppm correspond to the anomeric protons of the monosaccharide components of the PS, and these signals often serve as signatures for differentiating complex carbohydrate structures. The signals between 4.4 and 3.2 ppm are due to protons attached to C2-C6 and are poorly resolved due to the overlapping chemical shifts. The pattern of these signals, nonetheless, also reveals the similarities and differences between PSs. The signals at ~2.1 ppm, resulting from the methyl protons of N-acteyl or O-acetyl groups, clearly show the different degree of acetylation of each PS. In addition, the signals at 1.3 ppm that arise from the C-6 methyl protons of rhamnose are indicative of difference or similarity of the rhamnose component of the PS. Overall, the NMR spectral patterns revealed that the PSs from *E. faecium *B210860 and *E. faecalis *strains 324057B, type II, type V, and R19001 are different. However, the PSs from *E. faecalis *strains type V and 68114 have very similar structure (Fig. 2, bottom two figures). It is possible, in fact, that these two PSs are identical. The structural elucidation of these PSs is quite complicated and is part of an ongoing study.

### Immunological characterization of heteroglycans

To verify the antigenic specificity of the six heteroglycans, we obtained polyclonal antibodies against the heteroglycan PSs from *E. faecium *B210860 and *E. faecalis *type V. Because these PSs are not immunogenic in its native form, they were conjugated to tetanus toxoid. Both conjugates induced high titers of specific antibodies against the PSs. Therefore, the immunogenicity of these PSs can be significantly enhanced by conjugation to immunogenic protein carriers. If their specific antibodies are proven protective in future investigation, these PSs will become promising antigen candidates for enterococcal vaccines.

**Figure 3:**
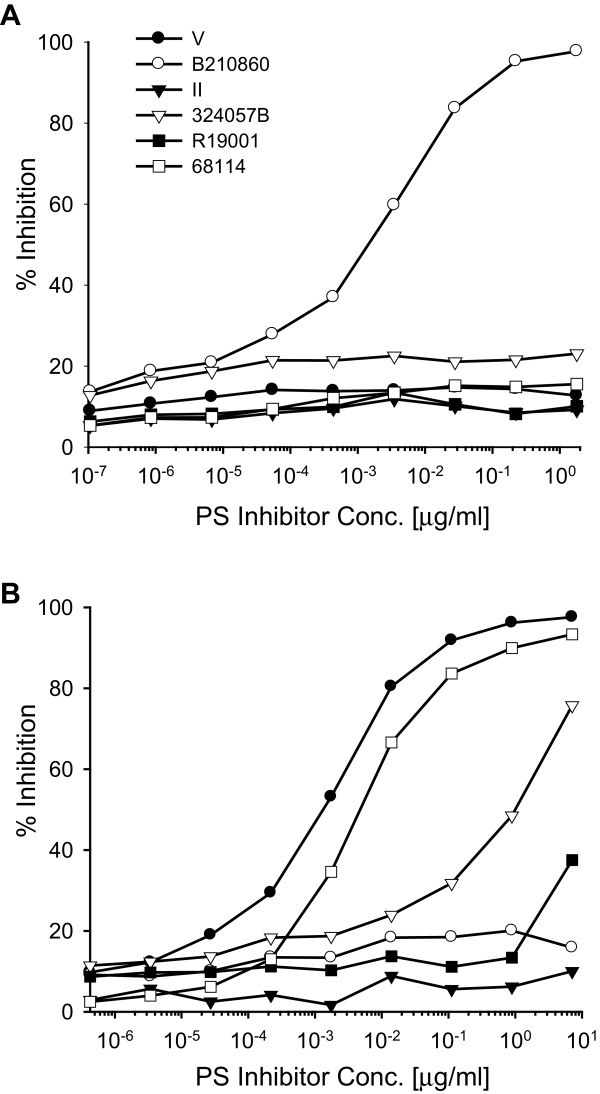
**Antigenic specificity of enterococcal heteroglycans**. Competitive ELISA inhibition of the binding between *E. faecium *B210860 PS and its specific antibodies (A) and inhibition of the binding between *E. faecalis *type V PS and its specific antibodies (B). Starting dilution of inhibitors were 10 times the coating concentrations.

The antisera specific for PSs of *E. faecium *B210860 or *E. faecalis *type V were used to probe the antigenic specificity of PSs from different enterococcal strains. The ability to inhibit the binding between these PSs and their specific antibodies by each of the six PSs was determined by competitive ELISA inhibition. As shown in Figure 3A, the results of the ELISA study indicate that the PS from the vancomycin-resistant *E. faecium *B210860 is significantly different from *E. faecalis *PSs. The binding between *E. faecium *B210860 PS and its antisera was only completely inhibited by itself. PSs from *E. faecalis *type II, 68114, and R19001 did not inhibit this binding even at high PS concentrations. Some batches of PSs from *E. faecalis *324057B and type V displayed limited inhibition at high concentrations.

On the other hand, the binding between *E. faecalis *type V PS and its specific sera could be inhibited to various percentages by the PS from *E. faecalis *strain 68114, 324057B, and R19001 (Fig. 3B). *E. faecalis *type II PS showed minimal cross-reactivity with type V PS, supporting the contention that these two strains belong to different serotypes. PS from the vancomycin-resistant *E. faecium *B210860 did not show cross-reactivity with the type V PS, which was confirmed in both inhibition assays (Figs. 3A and 3B). PS from *E. faecalis *68114 is highly cross-reactive with PS type V (Fig. 3B). Thus, both inhibition experiments and NMR analysis revealed that PSs from *E. faecalis *type V and 68114 are highly similar in structure and antigenicity. It is possible that these two PSs are the same antigen and that the subtle difference is the result of minor contamination in these PS preparations.

**Figure 4:**
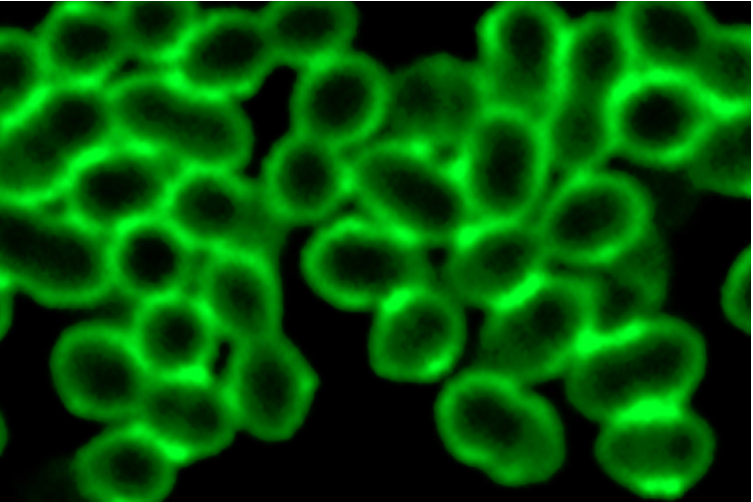
**Cell surface location of enterococcal heteroglycans**. Confocal micrograph of *E. faecalis *type V labeled with rabbit antisera specific for type V heteroglycan PS is show as an example. Note that these PSs are located at the cell surface.

This study reveals that all six enterococcal strains express three types of PSs, namely, a large dextran-like polyglucan, a medium-sized teichoic acid, and a heteroglycan with average molecular size of ~20 kDa. This result is in line with early findings that the PS gene cluster is widespread among *E. faecalis *strains and that *E. faecalis *produces several large and small PSs [13,22]. We are particularly interested in the heteroglycan PSs, because they are the most abundant PSs isolated from all six strains. We also confirmed that the heteroglycans are located at the cell surface by immunofluoresence spectroscopy (Fig. 4) although these heteroglycans could be part of the capsule, cell wall, or both.

## Discussion

Various capsular or cell wall enterococcal PSs have been reported. In 1963, Wicken and Baddiley identified an intracellular glycerol teichoic acid containing various glucose substituents as the group antigen for group D streptococci, to which enterococci previously belonged [25]. In 1973, Pazur et al. characterized an antigenic PS from *E. faecalis *consisting of a main chain of trisaccharide units joined with lactosyl and cellobiosyl side chains [26]. In 1967, Bleiweis et al. reported a type 1 antigen containing glucose, rhamnose, glucosamine, galactosamine, ribitol, and phosphorus [27]. In 1999, we characterized a teichoic acid consisting of glucose, glycerol, and phosphate common to several enterococcal strains [23,24]. Recently, Hancock et al. suggested that rhamnopolysaccharides consisting of rhamnose, glucose, galactose, glucosamine, and galactosamine are an invariant component of *E. faecalis *cells [22,28]. It is possible that the rhamnopolysaccharides and the heteroglycans in our study belong to the same category, e.g., they are both expressed at a similar cell surface location or serve similar functions in enterococcal physiology or pathogenesis.

## Conclusion

We have isolated and characterized the chemical and immunological properties of various PSs from six clinical strains of *E. faecium *and *E. faecalis*. Each strain produces large amounts of a complex heteroglycan consisting of glucose, galactose, rhamnose, N-acetyl-mannosamine, and N-acetyl-galactosamine. These PSs are not natively immunogenic but can be converted to potent immunogens by conjugation to protein carriers. Analysis by NMR spectroscopy and antigenicity analysis by competitive ELISA inhibition revealed that heteroglycan PSs from *E. faecium *B210860 and *E. faecalis *types II and V are distinct antigens. PSs from *E. faecalis *strains 324057B and R19001 share some epitopes with type V, whereas *E. faecalis *68114 PS is highly cross-reactive with type V PS. It is our hope that the heteroglycans may be used as the molecular basis for serotyping enterococcal strains and candidate antigens for the development of vaccines against multi-drug resistant enterococci.

## Methods

### Strain collection

A total of six clinical enterococcal strains were selected from a collection of clinical isolates. *E. faecalis *68114 and vancomycin-resistant *E. faecium *B210860 were isolated from patients at the Brigham and Women's Hospital in Boston [23]. *E. faecalis *324057B was isolated from a patient at the Beth Israel Deaconess Medical Center in Boston [23]. *E. faecalis *R19001 was an epidemic strain obtained from Boston Children's Hospital [29]. *E. faecalis *type II and type V strains were isolated from patients in various hospitals in Japan [30]. All isolates were grown in THB broth and 100-μl aliquots were stored in THB broth with 10% glycerol at -80°C.

### Bacterial culture

To obtain sufficient amounts of PSs for chemical and immunological analysis, 16-L batch cultures were prepared for each strain in 20-L carboys with Columbia dialysate broth supplemented with 5% glucose and 0.005% hemin. The dialysate broth was prepared by dialyzing Columbia broth (Difco Laboratories, Detroit, MI) in deionized water with a membrane of 10 kDa cut-off to remove molecules larger than 10 kDa. The pH of the growth medium were maintained between 7 and 7.2 by titration with 10 M NaOH through an automatic pH controller (Cole-Parmer Instrument, Vernon Hills, IL), and the fermentation was terminated when the pH of the growth medium stabilized without addition of NaOH. Bacteria were typically grown at 37°C for 16–24 h. Culture samples were examined for contamination by Gram staining.

### Isolation and purification of PSs

Bacterial cells were harvested by centrifugation at 4°C and washed in phosphate-buffered saline (PBS, 0.05 M phosphate, 0.15 M NaCl, pH 7). PSs were released from cell pellets by digestion with mutanolysin (50,000 units of enzyme per 100 g of wet cell) at 37°C for 16 h in 250 ml of buffer containing 40% sucrose, 30 mM phosphate (pH 7.0), 1 mM MgCl_2_, and 0.05% NaN_3_. After centrifugation, the supernatant was collected, dialyzed extensively against deionized water at 4°C, and lyophilized. The material was resuspended in a nuclease buffer (10 mM Tris, 10 mM CaCl2, and 10 mM MgCl2, pH 7.4) and treated with RNase A (0.5 mg/ml), DNase (0.1 mg/ml), and RNase T_1 _(5 μl/ml) at 37°C for 16 h, followed by digestion with proteinase K (0.1 mg/ml) at 56°C for 16 h. After centrifugation, the supernatant was collected, dialyzed extensively against deionized water; ethanol was added to a concentration of 30% and the mixture was chilled at 4°C for at least 6 hours to precipitate contaminating nucleic acids and proteins. After centrifugation, more ethanol was added to the supernatant to 80% concentration. PSs were precipitated at 4°C for 16 h and collected by centrifugation. Nuclease treatment and 30% ethanol precipitation were repeated until no significant amounts of proteins or nucleic acids were detected by UV absorption at 280 nm for protein contaminants and at 260 nm for nucleic acids and by ethidium bromide agarose gel for the absence of DNA.

The crude PS samples were further purified by size-exclusion and ion-exchange chromatography. The PS samples were first purified on Sephacryl S400 and S200 columns (Pharmacia Biotech, Piscataway, NJ). Eluted fractions were monitored by refractive index to detect PS and by UV absorption at 280 nm to detect protein contaminants. The carbohydrate content of each fraction was verified by phenol sulfuric acid assay. Fractions were also examined for immunogenicity by immunoblotting with rabbit antisera raised against the bacteria. Fractions positive with any of these four detection methods were pooled, dialyzed against deionized water, and lyophilized. They were further purified on an anion-exchange Q Sepharose column (Pharmacia Biotech, NJ), eluting with a linear gradient of NaCl from 0 to 1 M in 50 mM Tris buffer (pH 8.0). Similarly, fractions positive in the phenol-sulfuric acid assay or immunoblot were pooled, dialyzed, and lyophilized. To eliminate possible contaminants which may give false structural information, the heteroglycans were further purified by Superdex 200 column and analyzed by the same detection methods above.

### Chemical structural analysis of PSs

All PS samples were analyzed by ^1^H nuclear magnetic resonance (NMR) spectroscopy. Sample preparation consisted of 1 mg dissolved in 0.7 ml of D_2_O. The spectra were acquired on a Varian Unity 500 spectrometer at 60°C. ^1^H chemical shifts were referenced to the HDO resonance at 4.45 ppm. The water signal was suppressed by pre-saturation during data acquisition.

Monosaccharide compositions of PSs were determined by GC-MS analysis of the alditol acetate derivatives as described [24]. In brief, 0.05 mg of PS sample was completely hydrolyzed to monosaccharide components with 0.5 ml of 2 M trifluoroacetic acid at 110°C for 2 h, followed by reduction to their corresponding alditols with 5 mg of NaBH_4 _at room temperature for 2 h. Excess NaBH_4 _was removed by addition of 2 M HCl and drying under a stream of nitrogen, followed by addition of 0.5 ml of MeOH and drying under nitrogen. The alditols were converted to peracetates by treatment with 1 ml of 1:1 (vol.) mixture of acetic anhydride/pyridine at room temperature for 2 h. The alditol acetate derivatives were subjected to GC-MS analysis with a DB17 column on a Hewlett-Packard 6890/5973 GC-MS instrument. The GC run was started at 180°C, held for 2 min, and increased to 260°C by 2°C/min, and held at 260°C for 20 min. Standard alditol actetates corresponding to rhamnose, glucose, galactose, mannose, N-acetyl-glucosamine, N-acetyl-galactosamine, N-acetyl-mannosamine, and other typical monosaccharides were used for comparison in the GC-MS analysis.

### Preparation of antisera specific for bacterial cells or PSs

Antisera to whole bacterial cells of *E. faecalis *types II and V and *E. faecium *B210860 were prepared. Bacteria were grown in 500 ml of Todd-Hewitt broth at 37°C until mid-log phase with OD_650 _of 0.4–0.5. Bacterial cells were harvested by centrifugation, washed with PBS, and fixed with 0.1% formalin at room temperature for 30 min. Three New Zealand white female rabbits, 1.5 kg, were used for immunization with the three strains of enterococcal cells. Rabbits were immunized intravenously with 0.1, 0.3, and 0.5 ml of 7 × 10^9 ^cfu/mL bacterial cells on day 1, 3, and 5, respectively. On the second, fourth, and fifth weeks, the rabbits was boosted with 0.5 ml of the bacterial cells three times a week. Immune sera were obtained one week after the last immunization and used for identification of naturally immunogenic PSs.

Antisera against the PSs from *E. faecium *B210860 and *E. faecalis *type V were prepared from rabbits immunized with conjugates of PS to tetanus toxoid. For the conjugate preparation, 2-mg samples of PSs were oxidized with 0.5 ml of 10 mM NaI_4 _at room temperature for 90 min, dialyzed, and lyophilized. The oxidized PS was reacted with 2 mg of tetanus toxoid in PBS with the addition of 10 mg of NaCNBH_3_. The conjugates were dialyzed and lyophilized. One New Zealand white rabbit, 1.5 kg, was immunized with each of the two conjugates. Rabbits were immunized three times by subcutaneous injection with 50 μg conjugate on weeks 1, 3, and 6. Complete Freund's adjuvant was used in the primary injection and incomplete Freund's adjuvant was used in the booster injections. Immune sera were collected one week after the last immunization and used for assessing the antigenic specificity of PSs.

### Fluorescence confocal microscopy

*E. faecalis *type V and *E. faecium *B210860 were grown in glass-bottomed Petri dishes (MatTek, CA) with Todd-Hewitt broth to mid-log phase as determined by their growth curves. After being washed with PBS, bacterial cells were fixed with 1% paraformaldehyde at room temperature for 30 min. Cells were incubated with diluted (1:250) specific antisera at 37°C for 90 min, followed by incubation with Alexa Fluor 488-labeled goat-anti-rabbit IgG (Molecular Probes, CA) at 37°C for 90 min. Bacterial cells were washed and viewed by fluorescence confocal microscopy (Bio-Rad 1024MP system, Zeiss axiovert S100 cryton argon laser with 488 filter).

### Competitive ELISA inhibition

Nunc-Immuno microtiter plates were coated with 100 μL of the selected PS-bovine serum albumin (BSA) conjugate (1 mg/mL for *E. faecalis *Type V and 0.25 mg/mL for *E. faecium *B210860) at 30°C for 4 h. PS-BSA conjugates were prepared similarly to those of PS-tetanus toxoid as described above. Plates were then washed with 10 mM Tris buffer five times. In the meantime, a series of mixing solutions were prepared on a separate plate by adding inhibitory PSs in 2-fold serial dilution. The concentrations of the PS inhibitors started at a concentration of 2.5 mg/mL (10× coating-antigen) for *E. faecium *B210860 PS-BSA coated plates and at 10 mg/mL for *E. faecalis *Type V PS-BSA coated plates. The mixing plates were then incubated at 30°C for 2 h and washed five times, followed by the addition of 75 μL antisera to each well. For the competitive inhibition, 100-μL solution containing inhibitor and antisera in the mixing plate was transferred from each well to the antigen-coated plate. The plate was incubated at 4°C overnight, followed by addition of 100 μL alkaline phosphatase conjugated goat anti-rabbit IgG to each well. The plate was incubated at 37°C for 2 h, washed five times, and 100 μL of substrate solution was added containing 1 mg/mL of *p*-nitrophenyl-phosphate in bicarbonate buffer (35 mM sodium carbonate, 0.01% w/w MgCl_2_, 0.02% w/w sodium azide, pH 10). Absorption at 405 nm was recorded and plotted against molar concentrations of the inhibitor. Percentage of inhibition was calculated from the difference with and without inhibitor vs. control.

## Authors' contributions

C. T. Hsu conducted most of the experiments and assisted in experimental design and manuscript preparation. A. L. Ganong carried out ELISA and confocal microscopy experiments. B. Reinap prepared glycoconjugates. Z. Mourelatos assisted in bacterial culture and PS isolation. J. Huebner selected and provided enterococcal strains and the type-specific sera and assisted in the PS purification process and study design. J. Y. Wang designed experiments, performed structural analyses, analyzed the data, and wrote the manuscript. All authors approved the manuscript.
